# *Smarce1* and *Tensin 4* Are Putative Modulators of Corneoscleral Stiffness

**DOI:** 10.3389/fbioe.2021.596154

**Published:** 2021-02-05

**Authors:** Elizabeth M. Boazak, Rebecca King, Jiaxing Wang, Cassandra M. Chu, Aaron M. Toporek, Joseph M. Sherwood, Darryl R. Overby, Eldon E. Geisert, C. Ross Ethier

**Affiliations:** ^1^Wallace H. Coulter Department of Biomedical Engineering, Georgia Institute of Technology, Emory University, Atlanta, GA, United States; ^2^Department of Ophthalmology, Emory University, Atlanta, GA, United States; ^3^Department of Bioengineering, Imperial College London, London, United Kingdom; ^4^George W. Woodruff School of Mechanical Engineering, Georgia Institute of Technology, Atlanta, GA, United States

**Keywords:** *Smarce1*, *Tensin 4*, ocular compliance, scleral stiffness, corneal stiffness, mechanical properties, glaucoma, myopia

## Abstract

The biomechanical properties of the cornea and sclera are important in the onset and progression of multiple ocular pathologies and vary substantially between individuals, yet the source of this variation remains unknown. Here we identify genes putatively regulating corneoscleral biomechanical tissue properties by conducting high-fidelity ocular compliance measurements across the BXD recombinant inbred mouse set and performing quantitative trait analysis. We find seven cis-eQTLs and non-synonymous SNPs associating with ocular compliance, and show by RT-qPCR and immunolabeling that only two of the candidate genes, *Smarce1* and *Tns4*, showed significant expression in corneal and scleral tissues. Both have mechanistic potential to influence the development and/or regulation of tissue material properties. This work motivates further study of *Smarce1* and *Tns4* for their role(s) in ocular pathology involving the corneoscleral envelope as well as the development of novel mouse models of ocular pathophysiology, such as myopia and glaucoma.

## Introduction

Corneoscleral stiffness plays a role in multiple ocular pathologies, including glaucoma, myopia, and keratoconus, which together represent important sources of vision loss and blindness. The pathophysiologies underlying these conditions are poorly understood, and there exists a need for novel therapeutic strategies. For example, glaucoma is the leading cause of irreversible blindness worldwide (Cook and Foster, 2012). Elevated intraocular pressure (IOP), the primary risk factor for this condition (Leske et al., 2003), deforms optic nerve head tissues, likely accelerating vision loss. Scleral stiffness modulates such optic nerve head deformations (Sigal et al., 2005), and thus modulating scleral biomechanical properties has been suggested as a novel treatment for glaucoma. Myopia is expected to affect approximately half of the world's population by 2050 (Holden et al., 2016), and severe myopia is a risk factor for significant ocular pathologies, e.g., retinal detachment and glaucoma (Curtin and Karlin, 1970; Jacobi et al., 2005). Scleral extracellular matrix composition and biomechanical properties are thought to contribute to myopia risk (Jacobi et al., 2005); e.g., decorin (Rada et al., 2000; Siegwart and Norton, 2001) and lumican (Chakravarti et al., 2000; Austin et al., 2002) have been investigated in this context. Finally, although keratoconus is less prevalent than glaucoma and myopia (Gokhale, 2013), it clearly involves abnormal corneal biomechanics. Keratoconus is characterized by thinning of the central cornea and disorganization of collagen fibers (Daxer and Fratzl, 1997; Meek et al., 2005), leading to changes in corneal biomechanical properties and hence curvature/refraction (Rabinowitz, 1998). Keratoconus is thought to have both genetic and environmental factors (Mas Tur et al., 2017).

The identification of genes regulating corneoscleral biomechanics has potential to significantly advance our understanding of the aforementioned ocular diseases and may provide novel therapeutic targets for vision care. Here our specific goal was to identify candidate genes influencing corneoscleral stiffness, toward which end we measured ocular compliance in the BXD recombinant inbred mouse set, a powerful resource for quantitative trait locus (QTL) analysis (Geisert et al., 2009; Geisert and Williams, 2020). Ocular compliance, i.e., the ability of the eye to resist a change in internal pressure, is a useful and well-established surrogate measure of corneoscleral stiffness (Silver and Geyer, 2000; Pallikaris et al., 2005; Sherwood et al., 2019). We identified a number of candidate genomic loci associating with ocular compliance, and based on expression patterns, were able to identify two genes within these loci that are expressed in the corneoscleral envelope.

## Materials and Methods

### Animals

Male (*n* = 99) and female (*n* = 99) mice from the C57BL/6J and DBA/2J parental strains and from 20 BXD strains were used in this study. Breeding stock was ordered from Jackson Laboratories (Bar Harbor, ME); animals were bred and maintained at Emory University on a 12/12 h light/dark cycle. Animals were anesthetized with 100 mg/kg ketamine and 15 mg/kg xylazine and euthanized by decapitation with Mayo scissors. All mice used for ocular compliance measurements were between 77 and 100 days of age. The Institutional Animal Care and Use Committees at both Emory University and The Georgia Institute of Technology (GT) approved all studies, and all protocols adhered to the ARVO Statement for the Use of Animals in Ophthalmic and Vision Research.

### Ocular Compliance Measurements

Measurements of ocular compliance were carried out at Georgia Tech (GT). On a given day, 2–4 mouse heads were placed in 50 mL conical tubes filled with PBS and transported in a cooler on an ice pack from Emory to GT. Eyes were then enucleated using curved microscissors and tweezers under a dissecting microscope. As much of the extraocular tissues as possible were carefully cut away, while continually wetting the eye with phosphate buffered saline (PBS). Eyes were then weighed, placed in PBS in microcentrifuge tubes, and returned to the transport cooler until testing. Eyes of an individual pair were averaged, if masses for both eyes of the pair were available, prior to calculating whole-data-set and strain average masses. All testing was completed within 10 h of sacrifice.

Ocular compliance was measured using the iPerfusion system, as previously described (Sherwood et al., 2016, 2019). Briefly, the testing setup consisted of an adjustable pressure reservoir connected in series with a glass capillary (to add resistance to the system, improving measurement resolution), a flow sensor, a fluid manifold system (to enable calibration and testing procedures), a differential pressure sensor, and a glass needle (to cannulate the anterior chamber of the eye). Eyes were placed on a custom 3D printed holder in the eye bath that allowed for free expansion of the eye and facilitated cannulation without the need for application of glue. A series of increasing and decreasing changes in pressure were applied to an eye, enabling the measurement of ϕ as described in Sherwood et al. (2019) and below.

We were concerned that post-mortem duration could introduce unwanted variability into our measurements. Scleral material properties are not expected (Girard et al., 2007) to change significantly within 3 days of tissue storage at 4°C; however, corneal material properties would be expected to change due to loss of corneal epithelial function. We thus evaluated the effect of up to 10 h of eye storage duration on ocular compliance and outflow facility using C5Bl/6J mice (data not shown). Transport of whole heads submerged in PBS in a cooler containing an ice pack, followed by enucleation and eye preparation at GT, were determined to produce more consistent ocular compliance and outflow facility values than transporting enucleated eyes. Storage of prepared eyes in the transport cooler for up to 10 h was determined to have no effect on ocular compliance measurements or outflow facility measurements, which are highly sensitive to cell viability within the trabecular meshwork. Thus, it is very likely that corneal epithelial cell viability, and hence corneal material properties, were well-conserved up to 10 h post-mortem, i.e., within the time over which measurements were made.

### iPerfusion Data Analysis

Flow and pressure data from ocular perfusions were analyzed with both “Volume Filling” and “Step Response” approaches, previously described in detail (Sherwood et al., 2019). Briefly, the Volume Filling analysis method consists of integrating the measured flow rate into the eye, minus the flow leaving the eye through aqueous humor outflow, over an individual pressure step to give the volume change of the eye for a given change in pressure. The Step Response method makes use of a lumped parameter model of the iPerfusion system and eye, for which an analytical solution can be written for pressure as a function of time in response to a step change in applied pressure. This solution depends on ocular compliance over a given step change in pressure (ϕ), a non-linearity term (γ), and several known parameters. The measured pressure data in response to each step change in pressure is fit by the analytical solution, with ϕ and γ used as fitting parameters.

With both methods, a series of ϕ values for multiple pressures are obtained. ϕ is a strong function of pressure, and the ϕ vs. pressure data is thus fit with the empirical expression

(1) $$\begin{array}{r} \phi \left(P\right)=\phi_{r}\left(\frac{P_{r,\phi }+\gamma }{P+\gamma }\right) \end{array}$$

from which a reference compliance (ϕ_*r*_) can be obtained at a selected reference pressure (*P*_*r*, ϕ_). As the ϕ vs. pressure relationship is non-linear, a reference pressure at which to compare ϕ across BXD strains is required. We selected a value of *P*_*r*, ϕ_ of 13 mmHg, the normal intraocular pressure of C57BL/6J mice (Savinova et al., 2001).

Compliance is highly dependent upon ocular volume, i.e., a larger eye will have a larger ocular compliance, all other factors being equal. According to the ocular pressure-volume relationship described by Purslow and Karwatowski (Sherwood et al., 2019) (Equation 2), ocular compliance depends approximately linearly on ocular volume. We wished to identify candidate genes associating with ocular compliance that were independent of eye size. Thus, we normalized the ϕ_*r*_ values obtained for each eye by eye volume, giving ϕ_*norm*_. Eye volume was calculated from the eye mass measured prior to cannulation, and the published value of eye density, 1.103 mg/μl (Wisard et al., 2010), assuming that eye density was constant across BXD strains.

Our data analysis differed very slightly from previously published methods (Sherwood et al., 2019) in that both ascending and descending pressure steps were used in the Volume Filling method. Inclusion of both increasing and decreasing pressure steps in the calculation of ϕ_*r*_, as computed with the Volume Filling method, resulted in a lower average standard error across all strains, as compared to using increasing pressure steps alone. Meanwhile, the inclusion of decreasing pressure steps in the calculation of ϕ_*r*_ via the Step Response method increased the average standard error across all strains. As such, and in keeping with previously published methods, only increasing pressure steps were used in the Step Response analysis.

In all cases, the ϕ_*norm*_ data were subjected to several quality control steps. First, eyes in the top 15th percentile of uncertainty on the calculated ϕ_*r*_ values were eliminated from the data set. This was established as an empirical quality cut-off based on a preliminary analysis of flow and pressure traces from a subset of the data by two graders. Perfusions with high initial spikes in flow rate post-cannulation and high acclimatization flow rates (indicating ocular damage or leakage) were identified to be of poor quality; we found that these data sets consistently were in the upper 15th percentile of uncertainty on ϕ_*r*_. Then, ϕ_*norm*_ values from the remaining “best eyes” of each pair (i.e., the eye of a pair with lower uncertainty) were compiled to produce a data set for each BXD and parental strain. Values within each strain-specific ϕ_*norm*_ data set were identified as outliers if they lay more than 1.5 interquartile ranges above or below the upper and lower quartiles, respectively, and eliminated. The remaining data points were averaged to obtain a value of ocular compliance for each strain (${\bar{\phi }}_{norm}$).

While QTL analysis may be carried out with even one sample per BXD strain, attempts were made to include *n* ≥ 8 eyes per BXD strain, as permitted by the breeding propensity of the animals. Environmental factors have been shown to significantly influence outflow facility (Reina-Torres et al., 2019), and may contribute significantly to variations in other ocular properties, such as ocular compliance, as well. The ability to resample members of individual BXD strains decreases the influence of environmental and experimental variance, providing for a more accurate assessment of genetic variance.

### Interval Mapping of the Volume-Normalized Compliance Phenotype

The volume-normalized compliance data (${\bar{\phi }}_{norm}$) were subjected to conventional quantitative trait locus (QTL) analysis using simple and composite interval mapping along with epistatic interactions. ${\bar{\phi }}_{norm}$ values for multiple BXD strains (17 and 22 with the volume filling and step response data sets, respectively) were used to generate genome-wide interval maps, available on GeneNetwork.org. Genotype was regressed against each trait using the Haley-Knott equations implemented in the WebQTL module of GeneNetwork (Carlborg et al., 2005; Chesler et al., 2005). Empirical significance thresholds of linkage were determined by permutations (Churchill and Doerge, 1994). Phenotypes were correlated with gene expression data for the whole eye (Geisert et al., 2009; King et al., 2015).

### RNA Isolation and PCR

To validate the mRNA expression of potential candidate genes identified by QTL analysis, cornea, sclera and whole-eye tissue were isolated from C57BL/6J mice (70 days of age) and stored in Hank's Balanced Salt Solution with RiboLock (Thermo Scientific, Waltham MA) at −80°C. Each sample comprised four corneas (from two mice), four scleras (from two mice) or one whole eye; three independent samples were prepared for each sample type. RNA was isolated on a Qiacube with the RNeasy mini kit (Qiagen, Hilden, Germany) according to the manufacturer's instructions, with additional on-column DNase1 treatment. RNA integrity was assessed using an Agilent Bioanalyzer 2100, and RIN scores for both pooled tissues were >8.0. A QuantiNova Reverse Transcription Kit (Qiagen, Hilden, Germany) was used to retrotranscribe RNA for all tissues.

Reverse transcription-quantitative polymerase chain reaction (RT-qPCR) was used to validate the mRNA expression level of candidate genes (*Smarce1, Tns4, Abca13, Krt40, Krt33b, Ankrd36*, and *Gm11939*) in cornea, sclera, and whole eye. Primers were designed with Primer BLAST-NCBI so that predicted PCR products were 100–200 bp long (Table 1). PCR reactions were carried out in triplicate in 10 μl reactions containing 5 μl of 2× QuantiTect SYBR Green PCR Master Mix (Qiagen, Cat # 204141 Hilden, Germany), 0.5 μl of forward primer (0.5 μM), 0.5 μl of reverse primer (0.5 μM), 2 μl of template cDNA (10 ng), and 2 μl of Nuclease-free H_2_O. PCR was performed with a 15 min incubation at 95°C, followed by 40 reaction cycles comprised of denaturation at 94°C for 15 s, annealing at 59°C for 30 s, and extension at 72°C for 30 s. The cycle threshold (Ct) values were normalized to the mouse housekeeping gene peptidylprolyl isomerase A (*Ppia*) to generate Delta Ct values (Δ*Ct*) for each gene. Fold change (*FC*) in gene expression was calculated using the formula *FC* = 2^−Δ*ΔCt*^, where Δ*ΔCt* is the difference between the Δ*Ct* of tissue and the whole eye. Data are represented as mean ± standard error of the mean. Three biologically independent samples were used for statistical analysis using the Mann-Whitney-*U*-test.

**Table 1:** Primers used for RT-qPCR validation.

**Gene**	**Sequence (5^**′**^->3^**′**^)**	
*Smarce1*	Forward	CCAGCACACCAGGGTTTGTG
	Reverse	GTAATGCCAGAGGACGCCGT
*Tns4*	Forward	CAGGCGAGGACAGCAGTGAC
	Reverse	GGCTCTGCACCTCCCAGTTC
*Abca13*	Forward	ACAAGAGGTTCGGGATGGCTG
	Reverse	CAGCCGAGTACACAGAGCCTC
*Krt40*	Forward	TGGTCTGCCCCGATTATCAGC
	Reverse	GGCCAGTGATGTCGTTCTCCA
*Krt33b*	Forward	GGTGTTGCTGGATGTCAAGGC
	Reverse	CAGCCAAAGGAATTGCAGGGTC
*Ankrd36*	Forward	ACAGCATAAAGAAGTGGCATGGGA
	Reverse	CACTCCACACTCCCACCACAT
*Gm11939*	Forward	CCGTATCGTCCCCATTGGCC
	Reverse	TGAGCAGCCAACACGATGACA
*Ppia*		Mm_Ppia_1_SG QuantiTect Primer Assay

### Protein Expression: Immunohistology

Histological sample were obtained from C57BL/6J mice (70 days of age) that were deeply anesthetized (see above) and perfused through the heart with saline followed by 4% paraformaldehyde in phosphate buffer (pH 7.3). The eyes were embedded in paraffin as described by Sun et al. (2015). The eyes were serially dehydrated in diH_2_O and ethanol (EtOH) mixtures (50, 70, 90, and 95% EtOH), followed by two changes of 100% EtOH, 50% EtOH with 50% xylene, two changes of 100% xylene, and two changes of paraffin; all steps were 20 min. The eyes were then embedded in paraffin blocks. Ten micrometers sections were cut with a rotary microtome and mounted on glass slides. Paraffin was removed from the sections and the sections were rehydrated.

The sections were rinsed in PBS, and then placed in blocking buffer containing 2% donkey serum, 0.05% DMSO and 0.05% Triton X-100 for 30 min. The sections were incubated in primary antibodies overnight at 4° (*Smarce1*: Product ab228750, Abcam, Cambridge MA, 1:500; *Tns4*: Cat# 11580-1-AP, Proteintech IL, 1:50). After rinsing, the sections were incubated with secondary antibody conjugated to AlexaFluor-488 (donkey anti-rabbit, Jackson Immunoresearch Cat #711-545-152, Westgrove, PA, 1:1000) for 2 h at room temperature. The sections were then rinsed 3 × 15 min in PBS and counterstained with TO-PRO-3 iodide (T3605, Invitrogen, Eugene OR). The slides were flooded with Fluoromount-G (SouthernBiotech Cat # 0100-01, Birmingham, AL), and covered with a coverslip. Imaging was performed with a Nikon Eclipse TE2000-E (Melville, NY) confocal microscope and Nikon's EZ-C1 Software (Bronze Version, 3.91).

## Results

### Ocular Compliance Differs Appreciably Between BXD Strains

Ocular compliance is defined as $\phi =\frac{dV}{dP}$, where *V* is eye volume and *P* is IOP. ϕ reflects corneoscleral stiffness, and thus genomic loci associating with corneal and/or scleral stiffness will also associate with ϕ. We determined in ϕ in 198 mice from 22 BXD mouse strains from high-precision measurements of transient fluid ingress and pressure within enucleated eyes (see Methods). Specifically, compliance values were determined using two established independent methodologies, the so-called Volume Filling and Step Response approaches (Sherwood et al., 2019). Compliance values determined by the two methods differed slightly from one another, aligning with previous observations (Sherwood et al., 2019) and possibly reflecting a different emphasis on viscoelastic effects between the two methods (see Discussion). We thus carried out analyses using the two ocular compliance data sets in parallel.

**Figure 1:**
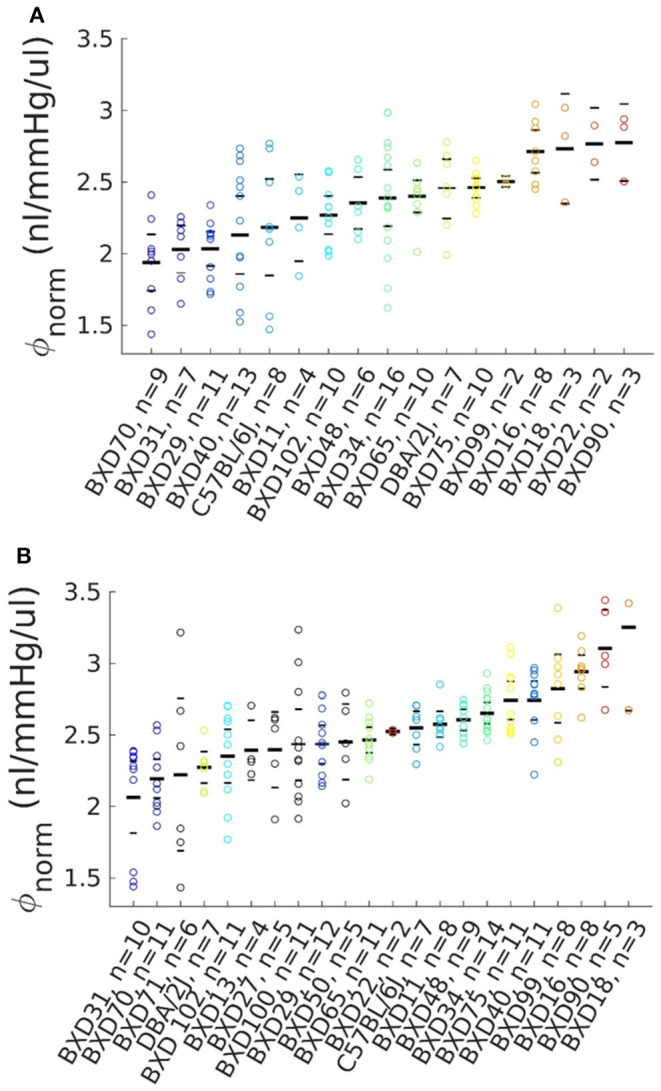
Volume-normalized ocular compliance from BXD mice. Bars mark the mean and limits of the 95% confidence interval for each strain, with each point representing one eye. Number of eyes is shown for each strain. **(A)** Compliance calculated using the Volume Filling method (see text), *n* = 129 eyes across 17 BXD strains. **(B)** Compliance calculated using the Step Response method (see text), *n* = 179 eyes across 22 BXD strains. The color scheme from strains included in the Volume Filling data set **(A)** is preserved in **(B)**, with additional strains shown in black.

Because ϕ depends strongly on IOP and ocular volume, further analysis used a normalized reference compliance, ϕ_*norm*_, computed from ϕ values by interpolating data to a common, physiologically-relevant reference pressure and dividing by ocular volume as determined from ocular mass measurements (see Methods). After applying elimination criteria and selecting one eye from each pair (see Methods), the Volume Filling data set comprised 129 eyes, and the Step Response data set comprised 179 eyes (Figure 1). Corresponding ocular compliance values, unnormalized by ocular mass, are shown in Supplementary Figures 1, 2. The ocular mass across all strains was 21.1 [20.9, 21.3] mg. The BXD70 strain had the lowest eye mass at 17.8 [16.9,18.8] mg, while the BXD11 strain had the highest at 23.3 [23.0, 23.7] mg. The ϕ_*norm*_ values for all eyes in each data set were 2.31 [2.24, 2.37] and 2.53 [2.47, 2.58] nl/mmHg/ul (mean [95% CI]), as obtained with the Volume Filling and Step Response methods, respectively.

As computed by the Volume Filling method, the strain with the lowest ${\bar{\phi }}_{norm}$ was BXD70, with an average value of 1.94 [1.74, 2.14] nl/mmHg/ul. BXD90 had the highest ${\bar{\phi }}_{norm}$ of 2.78 [2.51, 3.04] nl/mmHg/ul. The parental strains had intermediate ${\bar{\phi }}_{norm}$ values of 2.18 [1.85, 2.52] nl/mmHg/ul (B6) and 2.45 [2.25, 2.66] nl/mmHg/ul (D2). As computed with the Step Response method, the strain with the lowest ${\bar{\phi }}_{norm}$ was BXD31, with an average value of 2.06 [1.81, 2.32] nl/mmHg/ul, while BXD18 had the highest ${\bar{\phi }}_{norm}$ of 3.25 [2.67, 3.84] nl/mmHg/ul. The parental strains had intermediate ${\bar{\phi }}_{norm}$ of 2.55 [2.43, 2.66] nl/mmHg/ul (B6) and 2.27 [2.16, 2.38] nl/mmHg/ul (D2). The presence of BXD strains with higher and lower ${\bar{\phi }}_{norm}$ than the parental strains indicates that ϕ_*norm*_ is a complex trait. In both cases, significant differences in ${\bar{\phi }}_{norm}$ were observed between some strains (ANOVA, *p* ≤ 5 × 10^−8^), though significant differences are not required to effectively identify a QTL.

Heritability (*H*^2^) is the genetic variance (*V*_*g*_) of a trait as a fraction of the combined genetic and environmental (*V*_*e*_) variance of that trait, and was calculated for ϕ_*norm*_ by $H^{2}=\;\frac{V_{g}}{\left(V_{g}+V_{e}\right)}$. Genetic variance was approximated by the standard deviation of ${\bar{\phi }}_{norm}$ across strains, while environmental variance was estimated by calculating the mean of the standard deviations of ϕ_*norm*_ for all strains. *H*^2^ was 0.50 and 0.53, for data sets from the Volume Filling and Step Response data analysis approaches, respectively.

### QTL Analysis Revealed Highly Suggestive Loci Associating With Ocular Compliance

**Figure 2:**
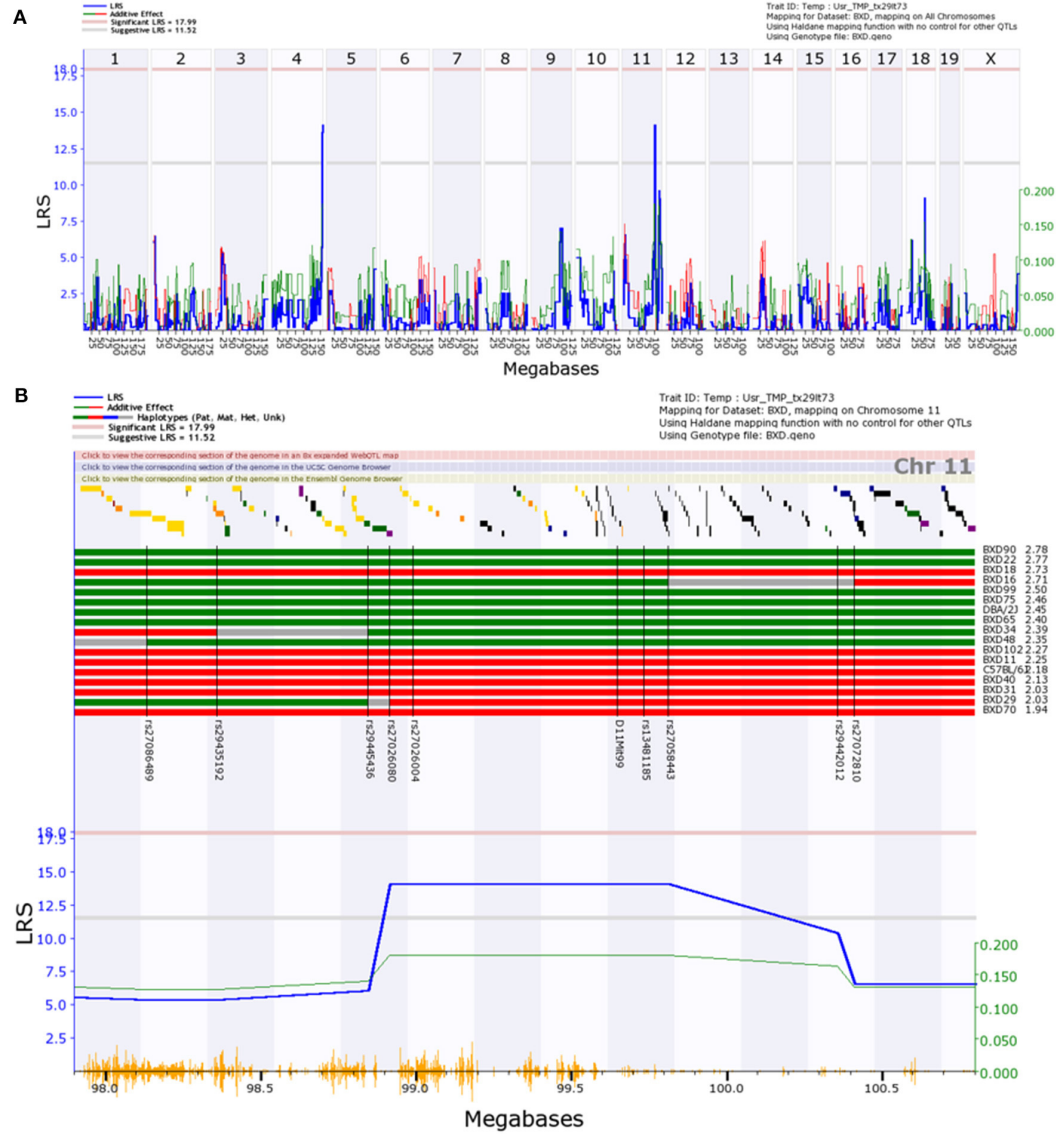
Interval map of normalized ocular compliance (${\bar{\phi }}_{norm}$) across the mouse genome, as determined with the Volume Filling data analysis method. The blue line in each panel represents the likelihood ratio statistic (LRS), a measure of the linkage between differences in the measured trait (${\bar{\phi }}_{norm}$) and genotype markers. Thresholds for significant (*p* < 0.05) and “suggestive” (*p* < 0.63) peaks are indicated with horizontal pink and gray lines (Abiola et al., 2003). Red and green peaks indicate the contributions of the B6 and D2 alleles, respectively. In **(A)**, a suggestive peak is observed on Chr11. In **(B)**, a magnified view of the region of interest is shown, accompanied by a haplotype map. B6 and D2 alleles are denoted by red and green bars, respectively, and are ordered from high to low ${\bar{\phi }}_{norm}$ values. Unmapped regions are shown in gray. Genomic markers used for interval mapping are labeled on the haplotype map and indicated with black vertical lines. Yellow vertical lines along the bottom of subplot **(B)** mark SNP locations.

We used bioinformatics tools (www.genenetwork.org) to generate genome-wide maps of trait linkage from the data shown in Figure 1. Genome-wide analysis of the Volume Filling data revealed a suggestive peak late on Chr11, bounded by the genetic markers rs29445436 (98.9 Mb) and rs27058443 (99.8 Mb), as well as a suggestive peak on Chr4 (Figure 2A). GeneNetwork identified no outliers in the Volume Filling data set. A magnified view of the highly suggestive region on Chr11 and the haplotype map (Figure 2B) show segregation of B6 and D2 alleles, with D2 alleles corresponding to greater ϕ_*norm*_. Peaks on Chr4 are notoriously unreliable (see Figure 13 in Geisert et al., 2009) and no genes with readily apparent biomechanical relevance were identified within the Chr4 locus; thus, we did not further consider this locus.

**Figure 3:**
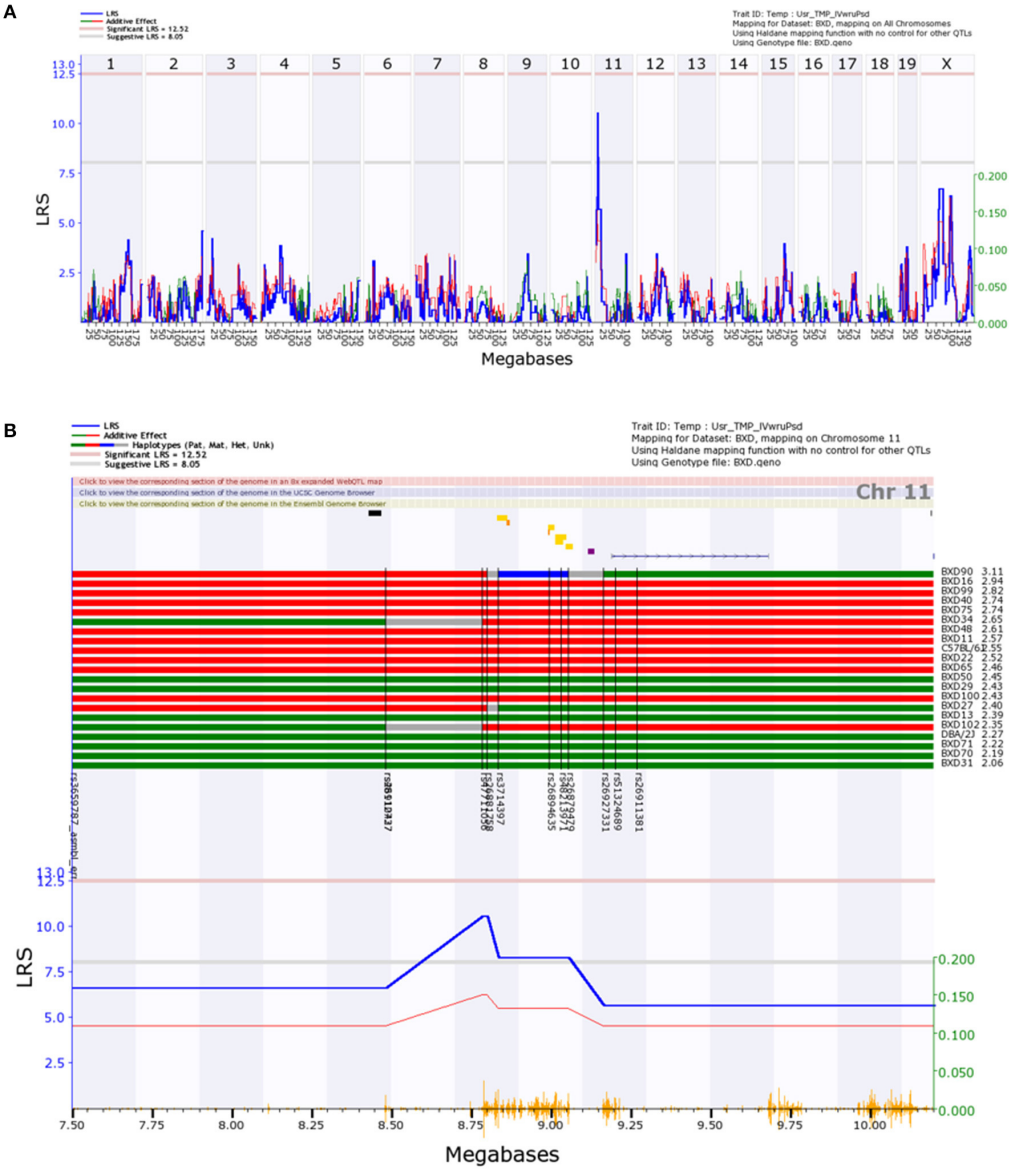
Interval map of normalized ocular compliance (${\bar{\phi }}_{norm}$) across the mouse genome, as determined with the Step Response data analysis method. Interpretation of Figure is as described in Figure 2.

Genome-wide analysis of the Step Response data (Figure 3A), excluding outliers, revealed a suggestive peak early on chr11 bounded by the genetic markers rs26910437 (8.5 Mb) and rs26927331 (9.2 Mb). GeneNetwork identified ϕ_*norm*_ for BXD18 as an outlier within the Step Response data set. A magnified view and haplotype map of the suggestive region (Figure 3B) shows that B6 alleles within this region increase ϕ_*norm*_, though the alleles do not segregate as consistently as within the locus identified via the Volume Filling method. The calculated effect size for the peak on chromosome 11 is 37%. This is an overestimate of the true effect size due to the use of BXD inbreed strains and the resampling of individual BXD strains that decrease the standard error of the mean, boosting the effective heritability (Belknap, 1998).

While our aim was not the identification of genes regulating eye size, it is possible that the assumption of equal eye density across strains and/or additional variability introduced from normalizing by measurements of eye volume could prevent the identification of other QTLs of interest. Thus, we also performed a genome-wide analysis of non-normalized ocular compliance ($\bar{\phi }$). Analysis of the non-normalized ocular compliance data as determined by the Volume Filling method revealed suggestive peaks late on chromosomes 4 and 11 (Supplementary Figure 3), in the same locations observed in the analysis of ${\bar{\phi }}_{norm}$, while no peaks were observed in the non-normalized ocular compliance data as determined by the Step Response method (Supplementary Figure 4).

### Candidate Genes for the Regulation of Ocular Compliance Reside in the Suggestive Loci

We next asked whether candidate genes for the regulation of ϕ_*norm*_ resided within the genomic loci identified via QTL analysis of both the Volume Filling and Step Response data sets. Genes within cis-expression QTLs (eQTLs), as well as non-synonymous single nucleotide polymorphisms (SNPs) within the identified loci have the potential to regulate ϕ_*norm*_. We thus searched the Whole Eye Database (Eye M430V2 (Sep08) RMA) hosted on GeneNetwork.org for genes residing within the ϕ_*norm*_ quantitative trait loci. Each search was performed over a region 1 Mb beyond the boundary genetic markers listed above, since BXD genotyping density allows for mapping with a precision of approximately ±1 Mb (Mulligan et al., 2017).

The search based on the Volume Filling results, performed for Chr11 from 97.9 to 100.8 Mb, identified 103 protein coding genes and three non-coding RNAs monitored by the Affymetrix M430V2 chip (Whole Eye Database hosted on GeneNetwork.org). When we examined RNAseq studies of the whole eye (Mustafi et al., 2012, 2016; Palczewska et al., 2016) all of the genes expressed in the RNAseq studies were also represented on the Affymetrix M430V2 chip with the exception of *Arl5c* (which does not contain any non-synonymous SNPs). Nine genes in the locus had expression likelihood ratio statistic (LRS) values above 17, corresponding to *p* ≤ 0.05, and were thus further investigated. Of these nine genes, seven were eliminated from the candidate list due to probes sets that bound either in intronic regions or to regions with significant SNPs between the B6 and D2 strains, i.e., the difference in gene expression between the parental strains was due to inappropriate probe interactions rather than mRNA expression levels. The remaining two candidate genes with cis-eQTLs were *Tns4* and *Smarce1*, in addition to three genes with disruptive non-synonymous SNPs (*GM11939, Krt40*, and *Krt33b*). For the Step Response data set, we examined Chr11 from 7.5 to 10.2 MB, identifying 10 genes, containing only one cis-eQTL (*Abca13*) and one disruptive non-synonymous SNP (*Ankrd36*). Each data set was evaluated for correlation with expression of candidate genes across BXD strains, and no significant correlations were identified. However, the non-synonymous SNPs of *Abca13* obviously correlate with the allelic segregation within the peak on chromosome 11 and if these disruptive changes in the gene affect protein function then we would expect protein function to correlate with the phenotype.

Even after normalization for ocular volume, ocular compliance is not an intrinsic property of the corneoscleral shell *per se*, e.g., it depends on corneoscleral shell thickness. It was therefore of interest to relate ocular compliance to an intrinsic property, namely the effective tensile elastic modulus of the corneoscleral shell, *E*_*effective*_, determined from the equation (Purslow and Karwatowski, 1996; Sherwood et al., 2019)

(2) $$\begin{array}{r} E_{effective}=\frac{3R}{4t}\left(\frac{V}{\phi }+P\right) \end{array}$$

where *R* is the radius of the eye, *t* the thickness of the corneoscleral envelope, and ϕ is the compliance of the eye at pressure *P*. We note that the above formula makes significant assumptions, namely that the corneoscleral shell is an isotropic, incompressible, homogenous, thin-walled sphere with a pressure-dependent elastic modulus. We calculated effective moduli at a reference pressure of 13 mmHg (Supplementary Figures 5, 6), using central corneal thickness (CCT) as a surrogate measure for the thickness of the corneoscleral envelope, and calculating eye radius from the volume-radius relationship for a sphere and the measured volume for each eye. The use of published central corneal thickness values (King et al., 2018) for corneoscleral thickness required the elimination of BXD48 and BXD70 from our data sets, since CCT values for these strains were not reported.

Mean effective tensile moduli for individual BXD strains ranged from 624 [590, 658] to 921 [840, 1,002] kPa, with an average value of 760 [735, 784] kPa for all strains, based on the Volume Filling compliance data. When based on the Step Response compliance data, mean effective tensile moduli ranged from 575 [457, 693] to 935 [800, 1,070] kPa, with a mean of 712 [693, 731] kPa. The tensile moduli of rodent cornea and sclera have been reported to range from ~2 to 5 MPa (Kling et al., 2012; Wang et al., 2012; Wu et al., 2013), and 0.4 to several MPa (depending on strain field) (Brown et al., 2020; Schwaner et al., 2020), respectively. Thus, our calculated effective moduli were comparable to previous reports.

Using the mean effective tensile elastic modulus for each strain, we again performed QTL analysis (data not shown). The Volume Filling data resulted in a suggestive peak in the same location on Chr11 as that described above. Additionally, a peak appeared in Chr9 from 88.5 to 94.8 Mb, but none of the genes with ocular expression in this locus and with a LRS > 17 were plausible candidates, since they all presented one or more of the probe binding issues discussed above. The Step Response data set gave rise to a suggestive peak on Chr14 from 40.1 to 55.1 Mb. Within this locus, 3 candidate genes were identified (*Cmtm5, Slc39a2*, and *Pnp2*), while other genes present in this locus were eliminated based on probe issues. Considering the extent of the assumptions required to determine a value for the intrinsic material properties of the eye, which itself is not homogenous, we elected to pursue only the candidate genes suggested from the normalized ocular compliance data. Nonetheless, *Cmtm5, Slc39a2*, and *Pnp2* may represent additional targets of interest, and data from additional BXD strains could help to narrow the peaks obtained from the effective elastic modulus QTL analysis.

### *Smarce1* and *Tns4* Are Transcriptionally Active in the Cornea and Sclera

**Figure 4:**
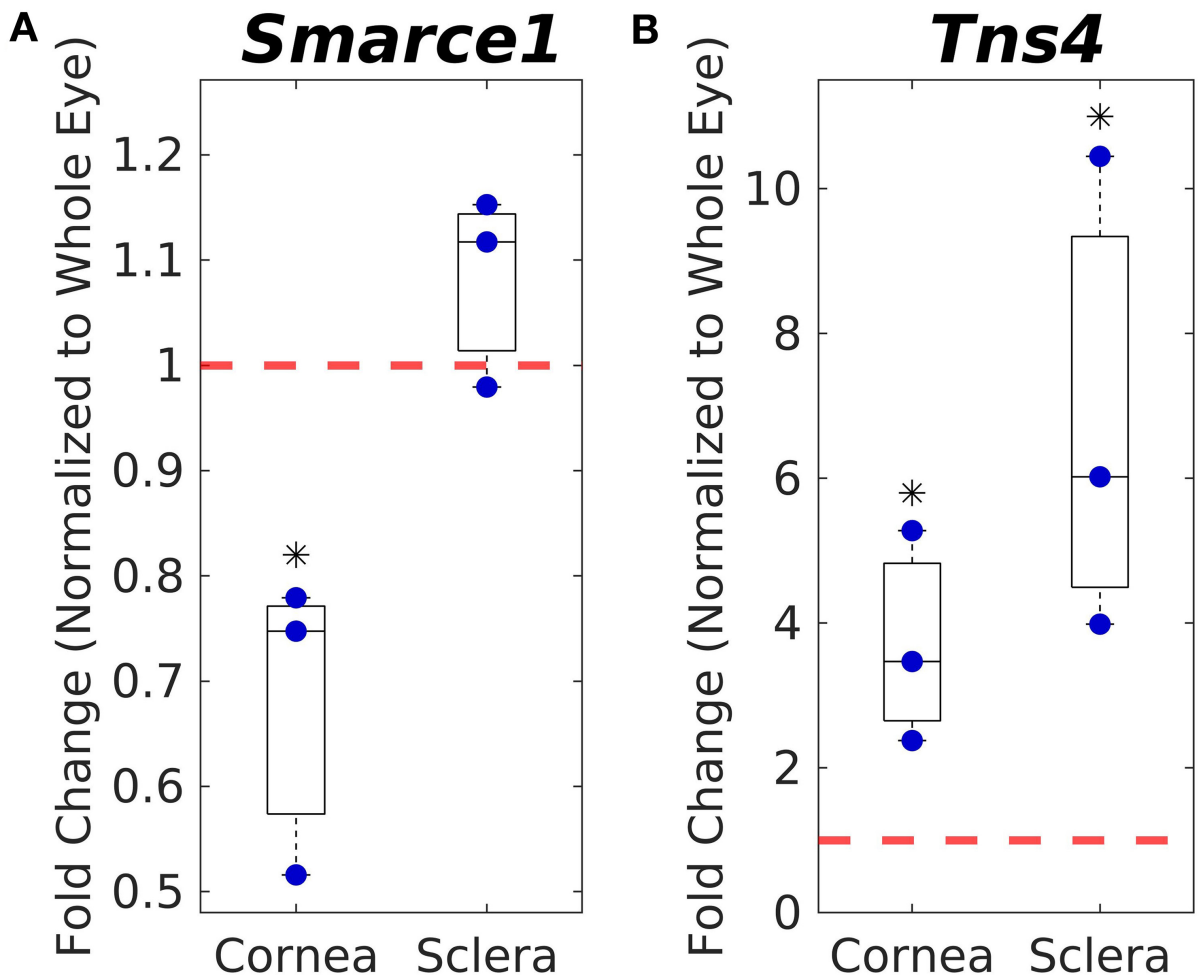
Corneal and scleral mRNA expression levels of *Smarce1* **(A)** and *Tns4* **(B)** normalized to whole eye expression levels (indicated by dashed red line), as measured by PCR. Asterisks indicate significant difference from whole eye expression levels.

We next asked whether transcripts for candidate genes identified from the ϕ_*norm*_ data set were present in the corneoscleral envelope. To address this question, we conducted reverse transcription-quantitative polymerase chain reaction (RT-qPCR) studies on cornea, sclera, and whole eyes. Among the 7 candidate genes identified above, only 2 genes were expressed at meaningful levels in the cornea and sclera (*Smarce1, Tns4*), with the other 5 genes (*Abca13, Krt40, Krt33b, Ankrd36*, and *Gm11939*) not detected after 40 cycles of amplification. The expression of *Smarce1* in the cornea was lower than in the whole eye (*p* = 0.05), while expression level in the sclera was slightly higher than in the whole eye, although this difference was not statistically significant (Figure 4). *Tns4* was significantly enriched in both the cornea (4-fold, *p* = 0.05) and the sclera (7-fold, *p* = 0.05), as compared with the whole eye (Figure 4). These results suggest that *Smarce1* and *Tns4* are viable candidate genes associating with normalized ocular compliance.

### *Smarce1* and *Tns4* Proteins Are Present in Cornea and Sclera

**Figure 5:**
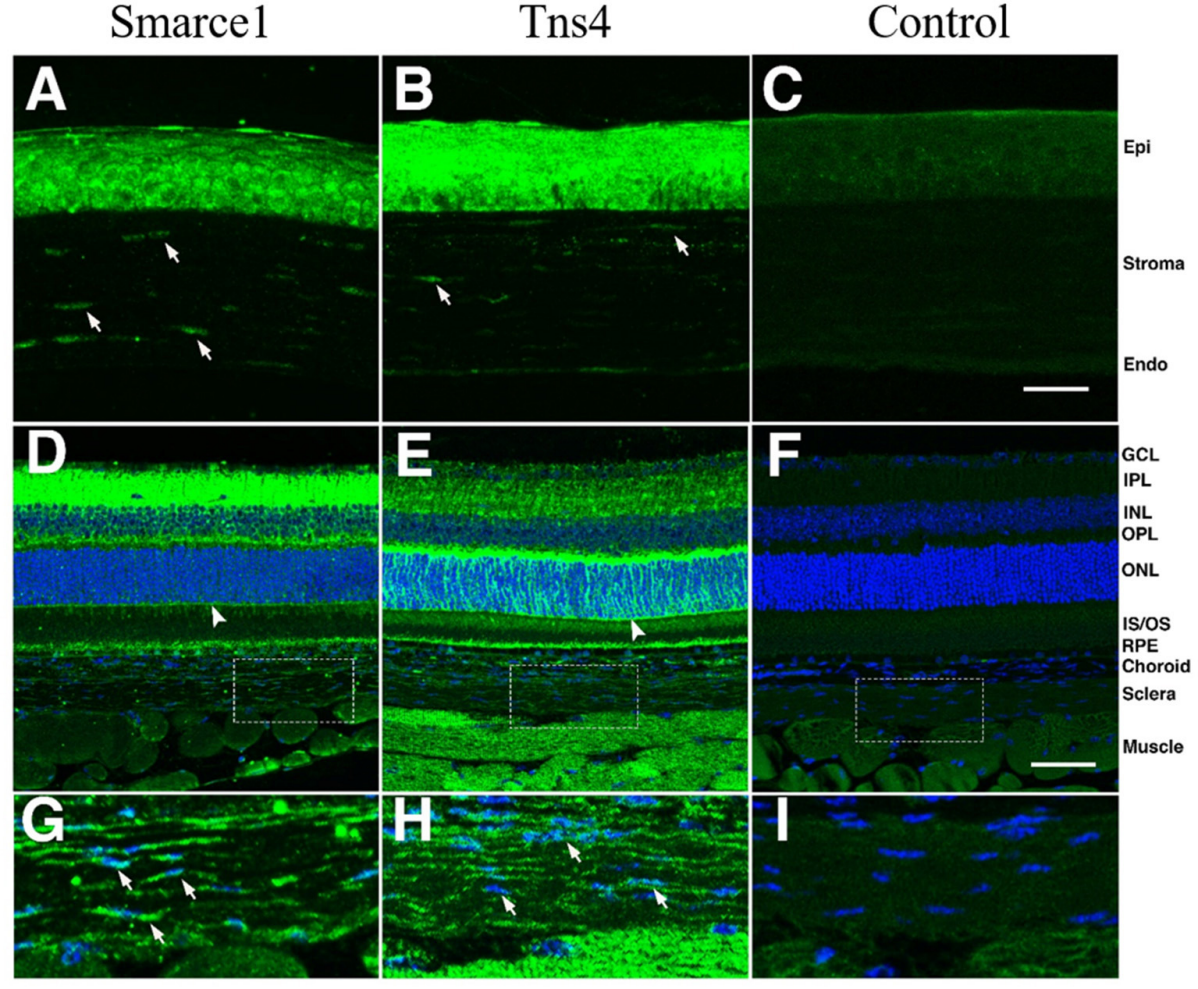
Staining patterns for *Smarce1* and *Tns4* protein in the mouse eye. Sections through the mouse eye were stained for *Smarce1* **(A,D,G)** and *Tns4* **(B,E,H)**, and the staining pattern was compared to similar sections stained with the secondary antibody only **(C,F,I)**. In the cornea **(A–C)** the epithelium and keratinocytes (arrows) were positive for *Smarce1* **(A)** and *Tns4* **(B)**. These structures were not stained in the secondary control. In the retina, both antibodies labeled cellular components, with positive staining of the external limiting membrane (arrowhead), suggesting that one cellular component recognized by the antibodies are Müller cells. In higher magnifications of the sclera **(G–I)**, staining of scleral keratinocytes can be observed (arrows) for both *Smarce1* and *Tns4*. The locations of the photographs of the sclera **(G–I)** are shown by boxes in **(D–F)**. The images in **(A–C)** are at the same magnification and the scale bar in **(C)** represents 25 μm. The images in **(D–F)** are at the same magnification and the scale bar in **(C)** represents 50 μm. The legend to the right indicates specific structures: corneal epithelium (Epi), corneal endothelium (Stroma, Endo), ganglion cell layer (GCL), inner plexiform layer (IPL), inner nuclear layer (INL), outer plexiform layer (OPL), outer nuclear layer (ONL), inner segments/outer segments (IS/OS), Choroid, Sclera, and Muscle.

To examine the distribution of *Smarce1* and *Tns4* in the eye, we stained sections though the C57BL/6J eye with antibodies directed against these two proteins (Figure 5). Both proteins were expressed in the cornea, the sclera and the retina. In the cornea, the epithelial layer was heavily labeled for *Smarce1* and *Tns4*, and there was labeling of the keratocytes within the corneal stroma. We also observed labeling in scleral fibroblasts. In both the corneal stroma and the sclera, *Smarce1* labeled cells more intensely than did *Tns4*. The retina also demonstrated a significant amount of labeling, labeling patterns depending on the target protein. Specifically, the heaviest *Tns4* labeling occurred in the inner plexiform layer (Figure 5D), while the heaviest *Smarce1* label was found in the outer plexiform layer (Figure 5E). The specific cellular components labeled by each antibody were not immediately obvious; however, in both cases it appeared that Müller cells were labeled, since the external limiting membrane was positive for both *Smarce1* and *Tns4*. These data demonstrate that, at least in the adult, *Smarce1* and *Tns4* are present in the cornea and sclera and thus have potential to influence corneoscleral material properties.

## Discussion

Our goal was to identify genes that could influence scleral stiffness, an important determinant of ocular biomechanics and certain ocular pathologies. Toward this end, we measured ocular compliance, an indirect measure of corneoscleral stiffness, in BXD recombinant inbred mice. The BXD mouse set is a powerful tool for QTL analysis, and allowed us to identify seven cis-eQTLs and non-synonymous SNPs with potential to influence ocular compliance. Message for two of the candidate genes, *Smarce1* and *Tns4*, was found in cornea and sclera, and their protein products were also present in these tissues. Further, the protein products of these genes have mechanistic potential to influence the development and or/regulation of tissue material properties (see below). Thus, *Smarce1* and/or *T* are excellent candidates for further investigation for their potential role(s) in affecting corneoscleral stiffness, of interest in glaucoma, myopia, and keratoconus.

It is mechanistically plausible that *Smarce1* and *Tensin* 4 influence corneal and/or scleral material properties. Tensins interact with actin filaments and β-integrins in focal adhesions (Lo, 2004), providing an important link between the extracellular matrix and the cytoskeleton. Tensins 1 and 3 are involved in matrix remodeling (Georgiadou et al., 2017), while *Tns4* (also known as C-terminal tensin-like, CTEN) localizes to focal adhesions, like its family members, but lacks the actin binding domain (Lo and Lo, 2002). Its upregulation is thought to promote cell motility and tumorigenicity (Lo, 2014), and can displace Tensin 3 from the β_1_ integrin cytoplasmic tail, disassembling actin fibers (Katz et al., 2007). In seeming contradiction, Kadmiel et al. (2016) showed that *Tns4* was upregulated in murine corneal epithelial cells following glucocorticoid (dexamethasone) treatment, which delayed wound healing via the attenuation of cell migration. While little is known about the role of *Tns4* in ocular tissues, and much remains to be studied regarding the role of tensins in mechanobiology, tensins are generally are thought to play a role in mechanotransduction (Georgiadou and Ivaska, 2017), making them an excellent candidate for the regulation of corneoscleral stiffness.

A putative mechanistic link between *Smarce1* and corneoscleral stiffness is less clear. *Smarce1* produces a subunit of SWI/SNF complexes, which are involved in chromatin remodeling. *Smarce1* is important for tissue-specific gene expression during development, and *Smarce1* mutation in zebrafish resulted in smaller eyes (Castillo-Robles et al., 2018). In addition to our demonstration that *Smarce1* is expressed in murine cornea and sclera, its expression has also been reported in human donor sclera (Young et al., 2004).

Determining the specific roles of these genes in influencing corneoscleral stiffness will require further study, and indeed a variety of experiments are immediately implied by our findings. For example, the role of our candidate genes in glaucoma, myopia, and keratoconus could be interrogated via the development of tissue-specific knockout/down mice. Further, an assessment of ocular compliance as a risk factor for glaucoma could be undertaken; specifically, ocular hypertension could be created in BXD strains with a range of ocular compliances, as identified here, and axon loss assessed and correlated with ocular compliance. Similar studies have been reported by Nguyen et al. (2013), exploiting natural variability in ocular stiffness between strains, but the use of the BXD mouse set may be a “cleaner” experiment that improves the power to determine the role that ocular compliance plays in axon loss. Similarly, a form-deprivation or lens-based mouse model of myopia could be implemented in BXD strains to investigate the link between ocular compliance and the rate of development of myopia. Additionally, *Tns4* and *Smarce1* signaling could be evaluated after form deprivation-induced myopia in wild type mice. Finally, a GWAS study would be of interest to explore the potential role of *Tns4* and *Smarce1* as risk factors in human presentations of glaucoma, myopia, and/or keratoconus.

In this regard, we examined the synteny map between mouse and human, finding that *TNS4* and *SMARCE1* were located on chromosome 17 at 38.6 Mb. Recent GWAS of human cornea (Iglesias et al., 2018; Shah et al., 2018) have identified loci associated with corneal thickness as well as disease states, such as keratoconus. However, neither of these studies identified *TNS4* and *SMARCE1* as potential candidate genes, nor were there any significant peaks near 38 Mb on chromosome 17. Two additional human GWAS studies examined risk factors associated with human myopia (Hysi et al., 2020) and glaucoma (Craig et al., 2020) and in neither of these studies were genetic risk factors identified that were in the vicinity of 38 Mb on chromosome 17. There are several possible explanations for this observation. First, it is possible that *TNS4* and *SMARCE1* play a role in molecular pathways associated with genes identified in human GWAS. Second, these GWAS data were generated for human cornea, for the regulation of eye growth associated with myopia and for a human disease (glaucoma). These studies do not directly address genes expressed in the human sclera that affect stiffness. It is possible that scleral stiffness contributes the majority of the compliance examined in our study and that the contribution of gene expression in human sclera to compliance may hold the key to understanding the relationship between *TNS4* and *SMARCE1* and human disease.

As described in the methods and results, pressure and flow data collected across a series of applied pressure steps were used to calculate ocular compliance. Two mathematical approaches for this calculation, termed the “Volume Filling” and “Step Response” methods, have been described previously in detail (Sherwood et al., 2019). Previous work showed good agreement between ocular compliance values obtained by the two methods, with higher measurement precision achieved with the Step Response method. However, since the previous comparison was made using a somewhat limited number of samples from a single strain of mouse, and since increased precision does not necessarily imply superior accuracy, we analyzed our larger data set with both methods. It is readily apparent from Figure 1 that the values for ϕ_*norm*_ reported here differ between the two analysis methods, resulting in different suggestive loci in the subsequent QTL analysis. Such a difference between the two calculation methods raises the question of whether one method is indeed more accurate, or whether the two methods capture different aspects of the mechanical response of the corneoscleral shell in response to changes in IOP. We further investigated this difference by performing Bland-Altman analysis (Supplementary Figure 7). Step Response ϕ_*norm*_ values were, on average, lower, but there did not appear to be any trend in the differences between the two methods. We hypothesize that the Step Response and Volume Filling methods capture different aspects of an eye's deformation under a change in IOP; specifically, the Volume Filling method better captures the viscoelastic creep that occurs in the corneoscleral shell over the entire time period of the applied pressure step, while the fitting of the analytical solution for the pressure response in time in the Step Response method is more influenced by the rapid tissue response immediately following the change in applied pressure. We suggest that this difference reflects different contributions of viscoelastic vs. elastic properties of the corneoscleral shell, respectively, and may also explain why the inclusion of decreasing pressure steps in the calculation of ϕ_*r*_ decreased average standard errors in the former, but increased standard errors in the latter (see Methods).

With the possibility that both Volume Filling and Step response data had the potential to reveal distinct candidate genes for the regulation of corneoscleral mechanical properties, both data sets were interrogated for the identification of quantitative trait loci and candidate genes, as well as correlation with identified genes and other parameters of interest. In both data sets, LRS values only reached “suggestive” levels. Measurement of additional BXD strains could increase the statistical significance of the identified peaks. In the case of the peak identified using the Volume Filling data set, there is a lack of additional genetic markers at which BXD strains could carry different parental alleles, needed to further narrow the region of interest. Thus, while additional measurements could result in reaching significance in the QTL analysis, the candidate genes identified would not change, barring the appearance of an additional suggestive or significant QTL. In the case of the Step Response data, additional measurements could narrow the QTL peak. However, as only one cis-eQTL and one non-synonymous SNP were identified from the Step Response data, we elected to move forward with evaluating tissue-specific expression of short list of candidate genes.

In order to identify possible confounding factors in our QTL analysis, we investigated whether ϕ_*norm*_ (determined from both the Volume Filling and Step Response data sets) was correlated with IOP, central corneal thickness (CCT), or eye size, all of which are available for BXD strains on genenetwork.org. In all cases, no significant correlations were identified. No correlation was expected with eye size, as dependence of the quantitative trait ϕ_*norm*_ on eye size was eliminated by normalization by eye volume. A lack of correlation between ϕ_*norm*_ and IOP is consistent with the hypothesis that scleral stiffness and/or ocular compliance could represent an additional risk factor for glaucoma. While elevated IOP is the primary risk factor for glaucoma, vision loss can occur at any level of IOP (Heijl et al., 2002; Leske et al., 2003; Leske, 2007), and a fraction of glaucoma patients present with normal IOP (Quigley et al., 2001; Iwase et al., 2004). Importantly, the lack of correlation between ϕ_*norm*_ and CCT, a highly heritable ocular trait (King et al., 2018) and phenotypic risk factor for glaucoma (Sng et al., 2017), indicates that: (i) ocular compliance reflects both scleral and corneal material properties (i.e., ϕ_*norm*_ is not governed disproportionately by corneal material properties), and/or (ii) that variations in corneal material properties do not directly compensate for changes in corneal thickness.

Finally, we also evaluated the correlation between ϕ_*r*_ (non-normalized) and our own ocular volume data (Supplementary Figure 8). The expected trend, i.e., greater eye volume corresponding to higher ocular compliance, was observed, though coefficients of determination were low with both the Volume Filling and Step Response data sets. Low coefficients of determination are consistent with the strain-dependent variations in corneoscleral material properties having a measurable impact on ocular compliance.

This work is subject to certain limitations. For example, ocular compliance measurements were made on post-mortem tissues, which could induce artifacts. However, prior to collection of the compliance data presented in this manuscript, multiple aspects of the compliance measurement methodology were optimized. For example, we determined that post-mortem time did not affect our measurements (see Methods). A second possible limitation is that we use ocular compliance, which reflects corneoscleral mechanical properties, as our phenotypic quantitative trait, as opposed to a tissue-specific quantitative trait. We suggest this is, however, actually a benefit: measurement of ocular compliance, unlike alternative methods [e.g., strip testing (Phillips and McBrien, 1995; Elsheikh and Anderson, 2005; Lari et al., 2012; Hatami-Marbini and Rahimi, 2014), compression (Battaglioli and Kamm, 1984; Mortazavi et al., 2009; Boazak et al., 2019)] evaluates the eye in its natural geometrical configuration. Further, ocular compliance measurements are faster and easier to carry out in small murine eyes as compared to alternatives (Myers et al., 2010; Hannon et al., 2019), and they do not require fixation of the eye to a mounting block, which can potentially introduce non-physiological boundary conditions. A third possible limitation was the use of eye mass to calculate eye volume, which in turn was used to calculate ϕ_*norm*_. Inconsistency in the removal of extraocular tissue, eye hydration, or IOP (which could be affected by enucleation technique) at the time of measurement may have slightly influenced the recorded eye mass; this concern was countered by the use of multiple samples per BXD strain, and by evaluation of unnormalized data (see Results, Supplementary Figures 3, 4).

In summary, we use a novel methodology to identify several genes putatively associating with corneoscleral biomechanical properties, *Smarce1* and *Tns4*. Further investigation of the role(s) of these genes in glaucoma, myopia and keratoconus is strongly indicated.

## Data Availability Statement

The raw data supporting the conclusions of this article will be made available by the authors, without undue reservation. Mean and standard error for key metrics reported here (normalized ocular compliance, ocular compliance, corneoscleral effective modulus, and eye weight) for each BXD strain assessed are available on genenetwork.org.

## Ethics Statement

The animal study was reviewed and approved by Institutional Animal Care and Use Committees at both Emory University and The Georgia Institute of Technology (GT).

## Author Contributions

EB, RK, JW, and CC conducted the experiments. EB, AT, JS, DO, EG, and CE analyzed the data. EB, JS, DO, EG, and CE wrote the manuscript. DO, EG, and CE conceived the study. All authors contributed to the article and approved the submitted version.

## Conflict of Interest

The authors declare that the research was conducted in the absence of any commercial or financial relationships that could be construed as a potential conflict of interest.
