# Supplementary material for: Smarce1 and Tensin 4 Are Putative Modulators of Corneoscleral Stiffness
Source: Front Bioeng Biotechnol. 2021 Feb 5;9:596154. doi: 10.3389/fbioe.2021.596154 (PMC7902041; doi:10.3389/fbioe.2021.596154)
Supplement: Supplementary file 1 [file Data_Sheet_1.docx]

Supplementary Material

**
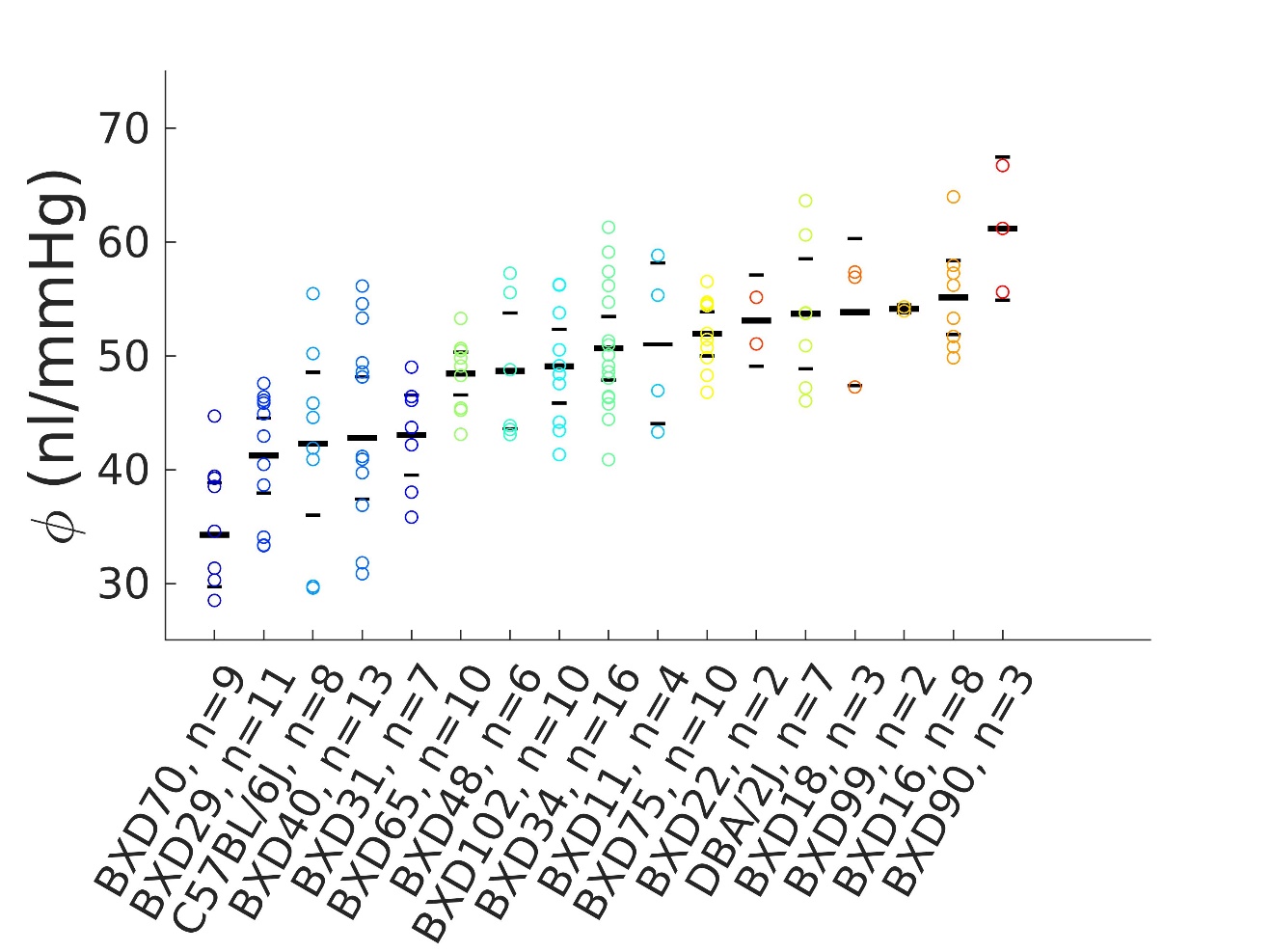
**

**Supplemental Figure 1:** Non-normalized ocular compliance from the same 129 eyes across 17 mouse strains shown in Fig 1A, as calculated with the Volume Filling method. Strains are ordered from low to high ocular compliance, with strain colors based on the normalized compliance values and color scheme shown in Fig 1. Bars mark the mean and limits of the 95% confidence interval for each strain, with each point representing one eye. Number of eyes is shown for each strain.

**
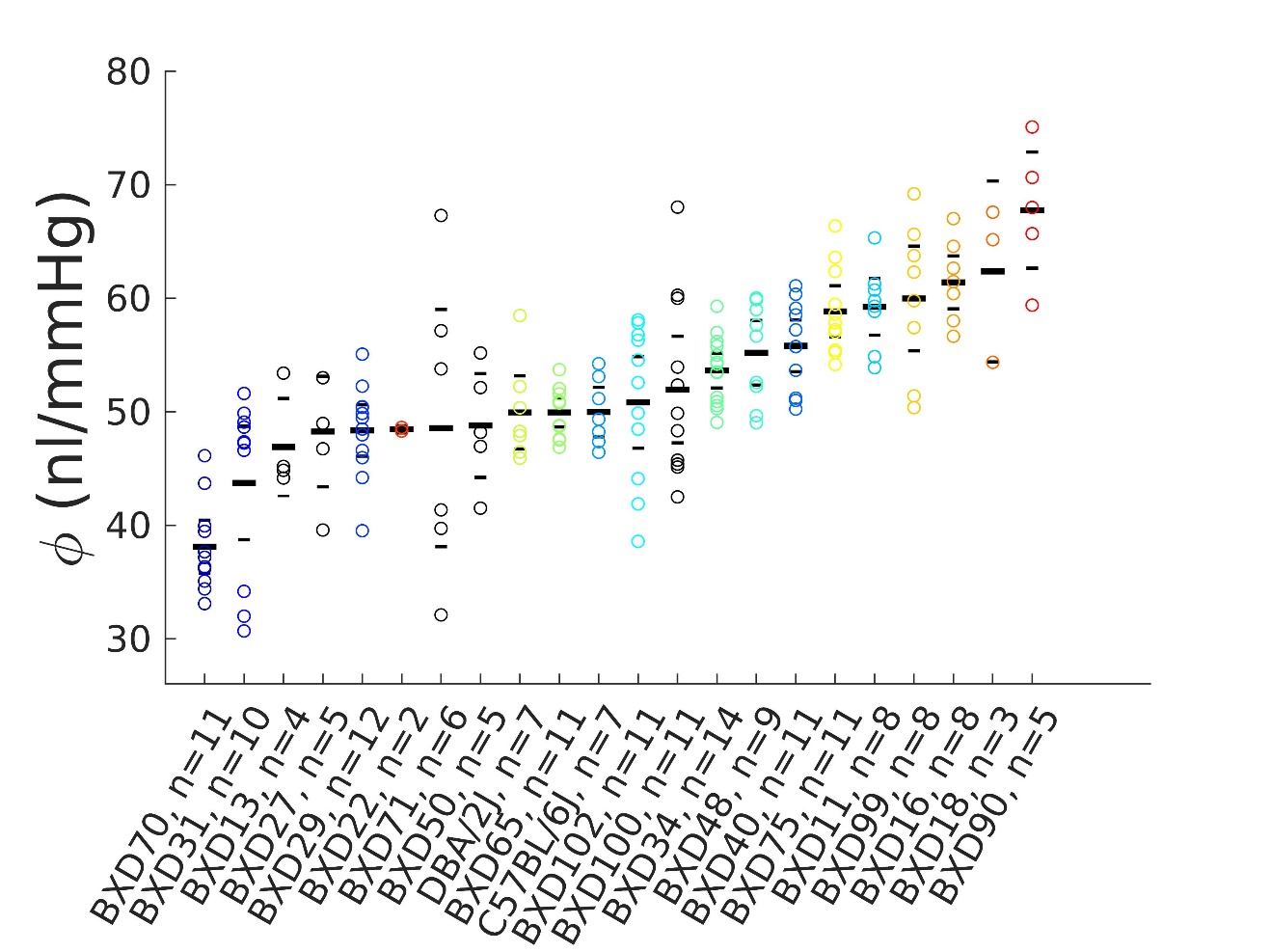
**

Supplemental Figure 2: Non-normalized ocular compliance from the same 179 eyes across 22 mouse strains shown in in Fig 1B, as calculated with the Step Response method. Strains are ordered from low to high ocular compliance, with the color scheme based on the normalized compliance values shown in Fig 1. Refer to Figure 1 for details of interpreting symbols.


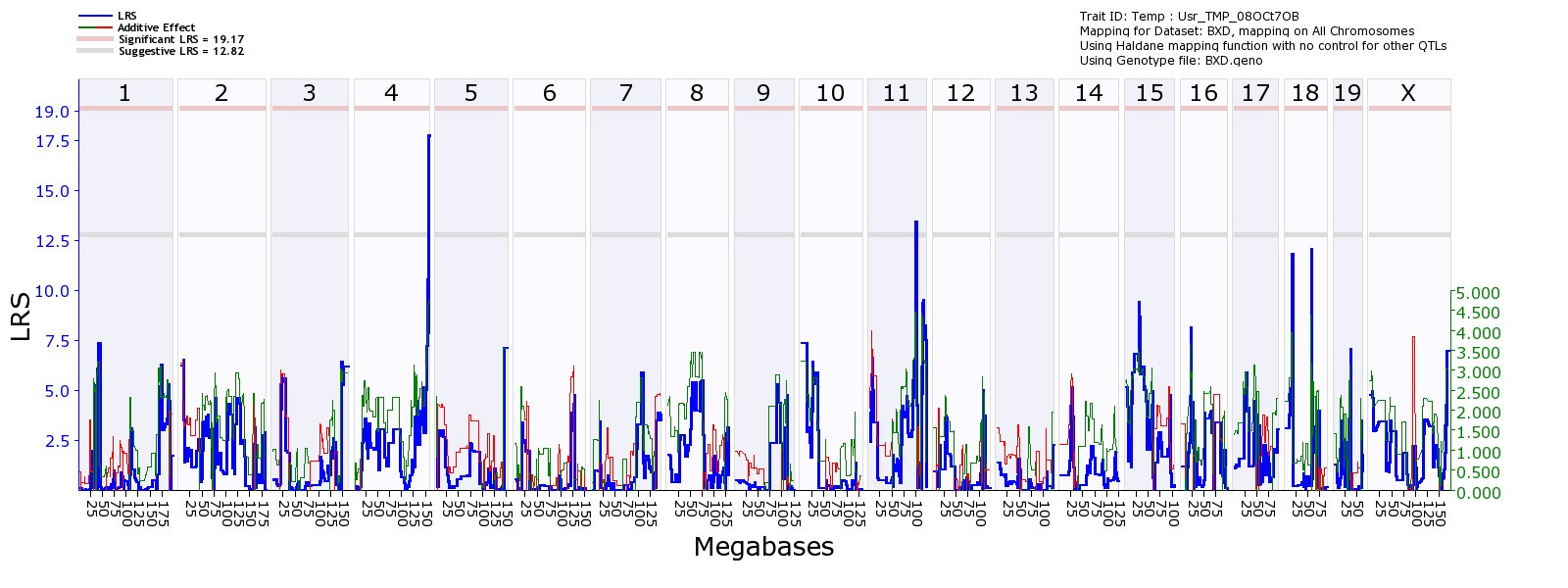


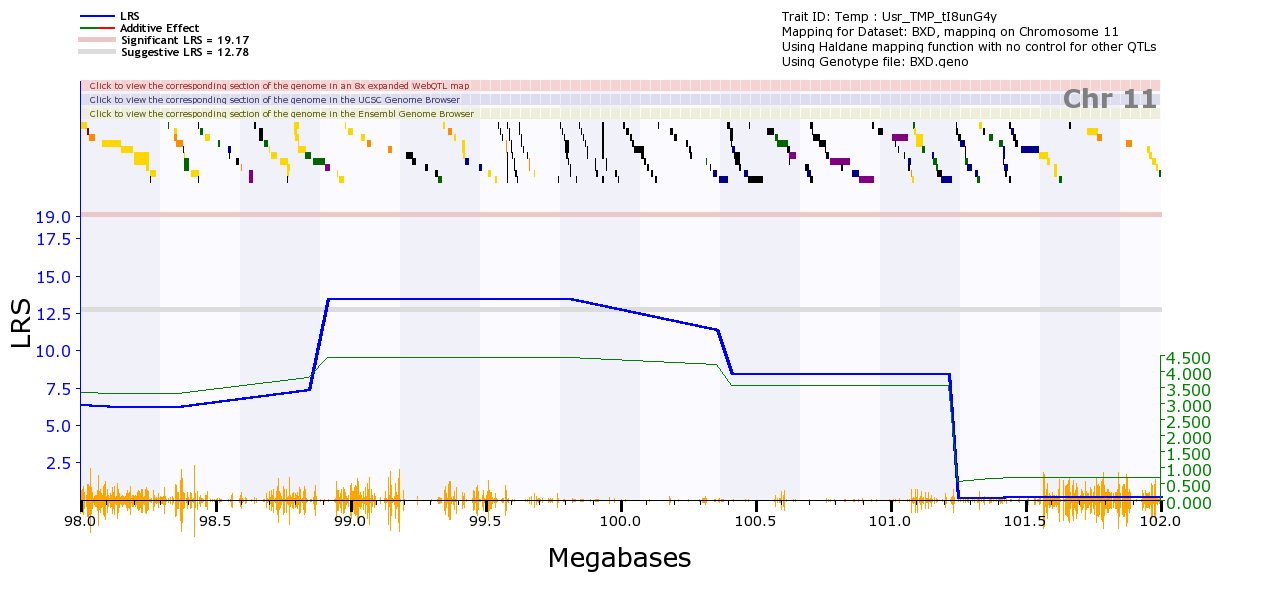
 **Supplemental Figure 3:** Interval map of non-normalized ocular compliance (𝜙) across the mouse genome, as determined with the Volume Filling method. The interpretation is as in Figures 2 and 3 in the paper. The top panel shows the entire mouse genome, with suggestive peaks late on chromosome 4 and late on chromosome 11, while the bottom panel shows a magnified view of the peak on Chr11.


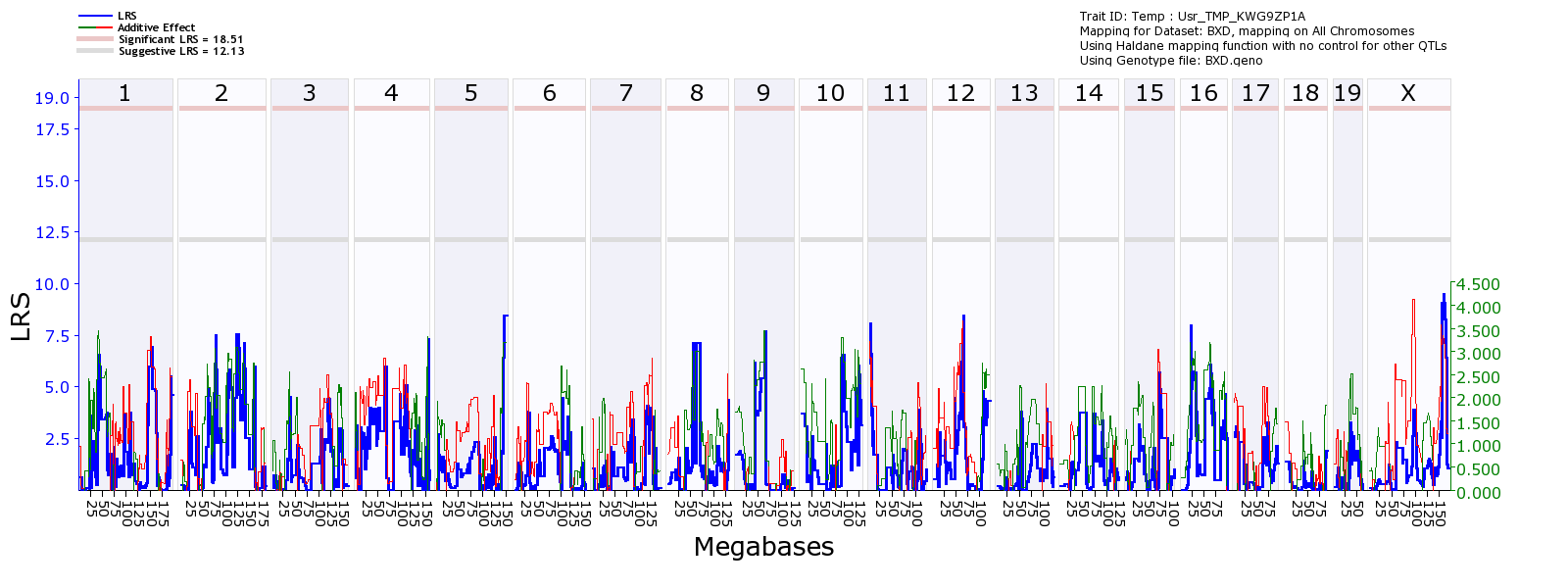


**Supplemental Figure 4**: Interval map of non-normalized ocular compliance (𝜙) across the mouse genome, as determined with the Step Response method. The interpretation is as in Figures 2A and 3A in the paper. No suggestive peaks are observed.

**
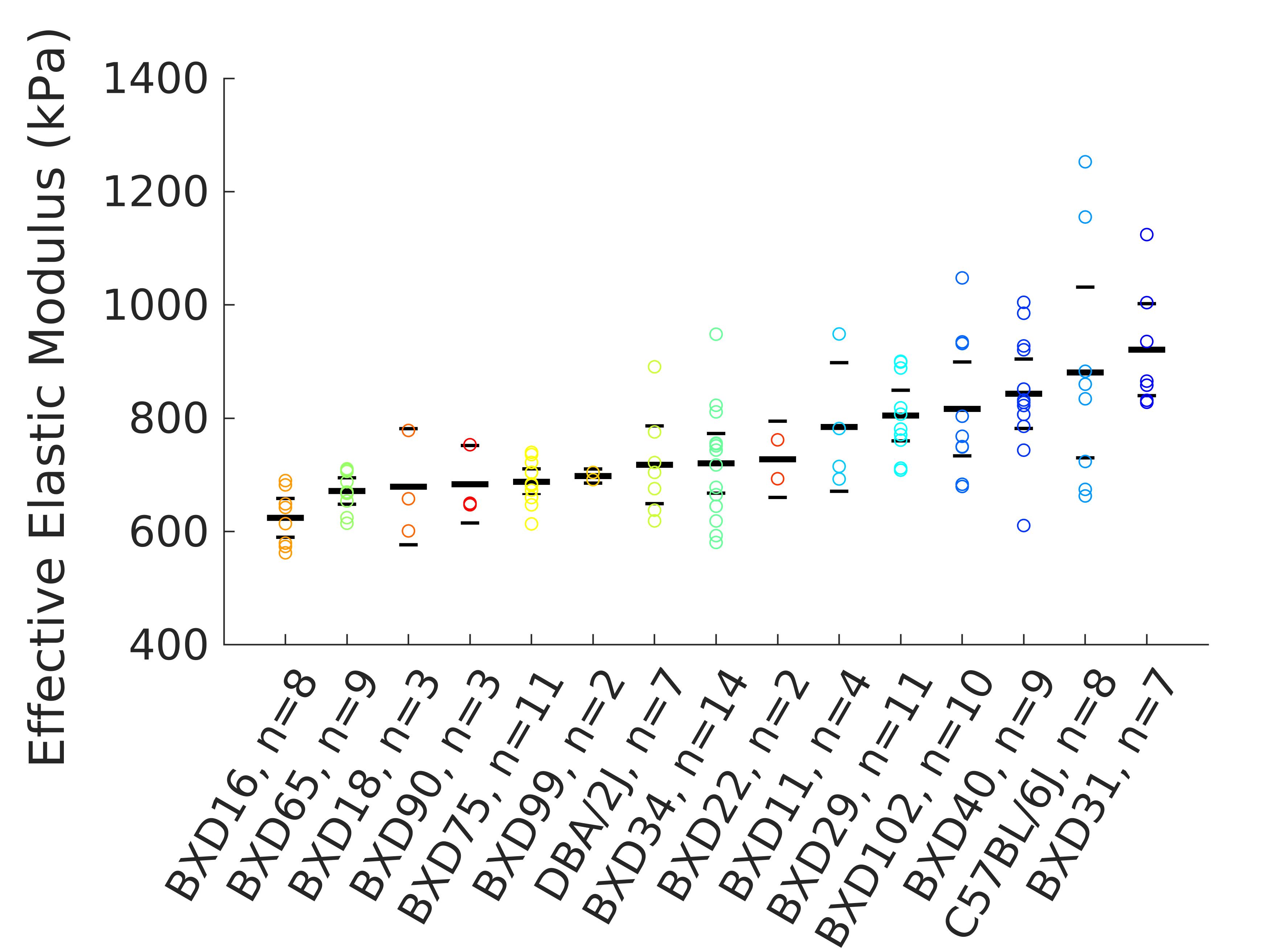
**

**Supplemental Figure 5:** Effective modulus, as calculated with the Volume Filling method. The same eyes from each pair were chosen, as shown in Figure 1A and Supplemental Figure 1, although the number of eyes included per strain varied based from previous figures based on which values were identified as outliers within each strain. Strains are ordered from low to high modulus, with strain colors based on the normalized compliance values and color scheme shown in all other scatter plots. Bars mark the mean and limits of the 95% confidence interval for each strain, with each point representing one eye. Number of eyes is shown for each strain.

**
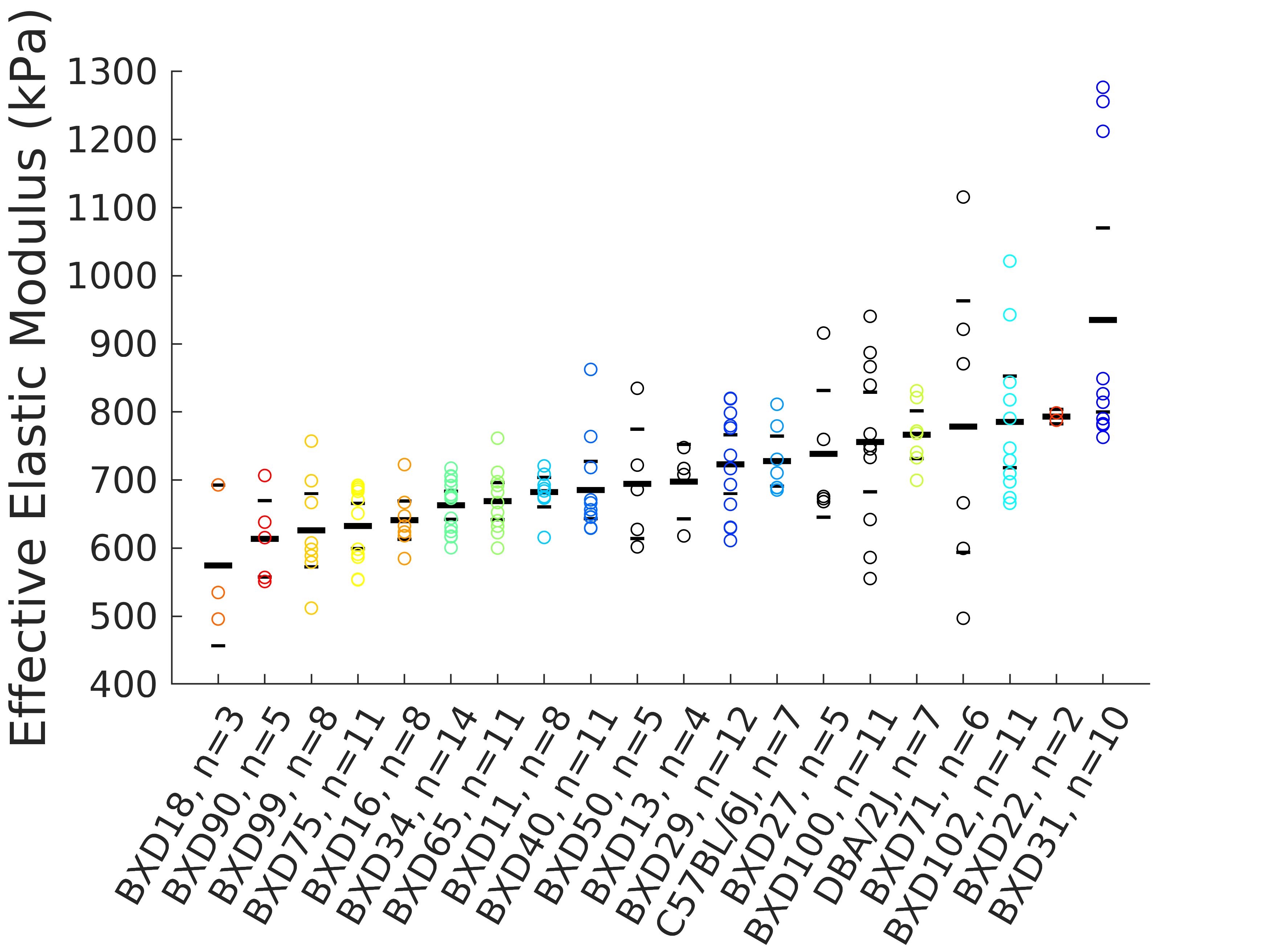
**

**Supplemental Figure 6:** Effective modulus, as calculated with the Step Response method. The same eyes from each pair were chosen, as shown in Figure 1B and Supplemental Figure 2, although the number of eyes included per strain varied based from previous figures based on which values were identified as outliers within each strain. Strains are ordered from low to high elastic modulus, with strain colors based on the normalized compliance values and color scheme shown in all other scatter plots. Bars mark the mean and limits of the 95% confidence interval for each strain, with each point representing one eye. Number of eyes is shown for each strain.


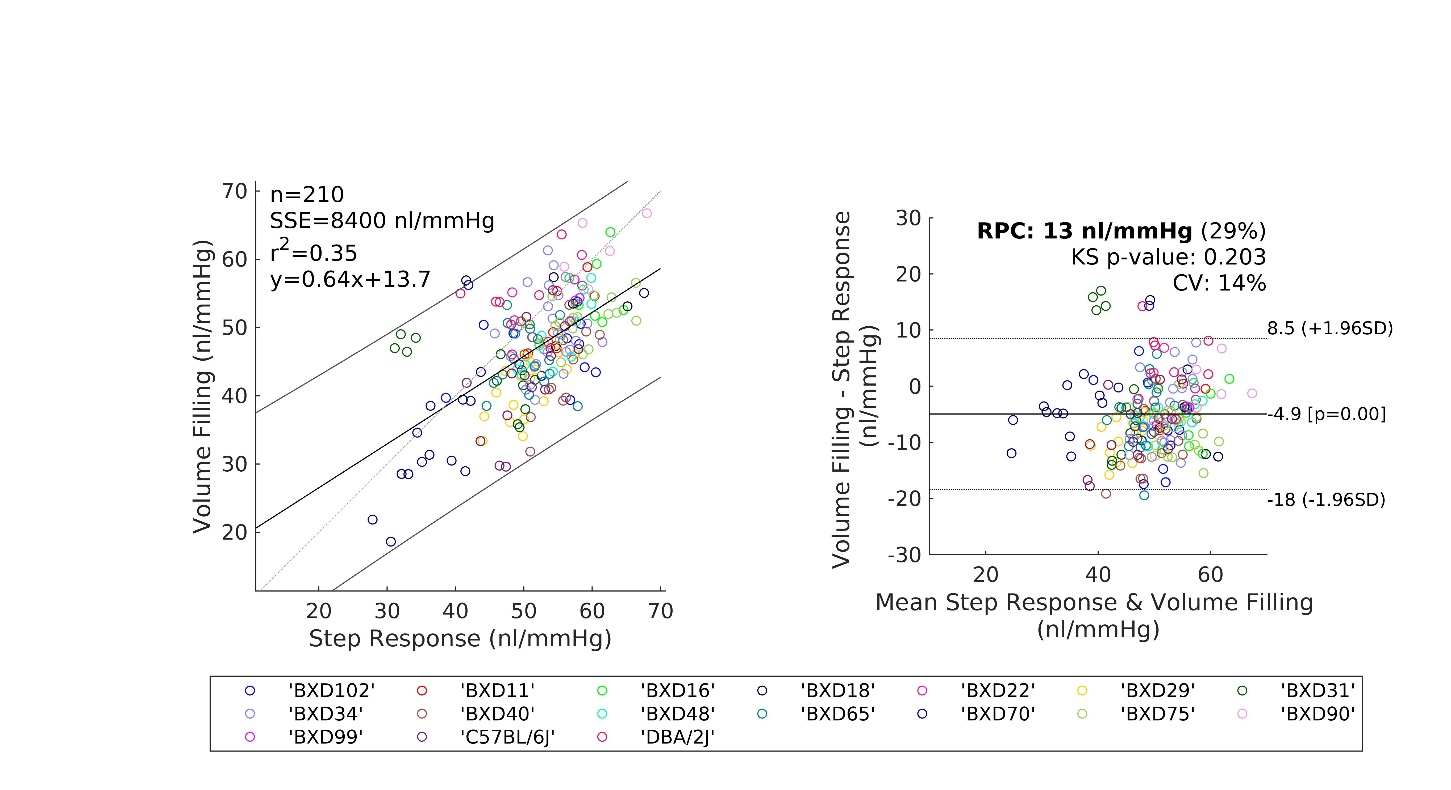


**B**

**A**

**Supplemental Figure 7:** Correlation (A) and Bland Altman (B) plots comparing ocular compliance for 210 individual eyes, as measured by the Step Response and Volume Filling methods. The repeatability coefficient (RPC) is equal to 1.96 times the standard deviation of the differences in compliance between the two methods, and is the distance the limits of agreement lie from the mean difference. This is the interval within which 95% of the Step Response and Volume Filling measurement differences lie. The p-value obtained using the Kolmogorov-Smirnov test (KS p-value) is >0.05, indicating that the data set follows a Gaussian distribution. The coefficient of variation (CV) gives the standard deviation of the mean Step Response and Volume Filling values as a percentage.


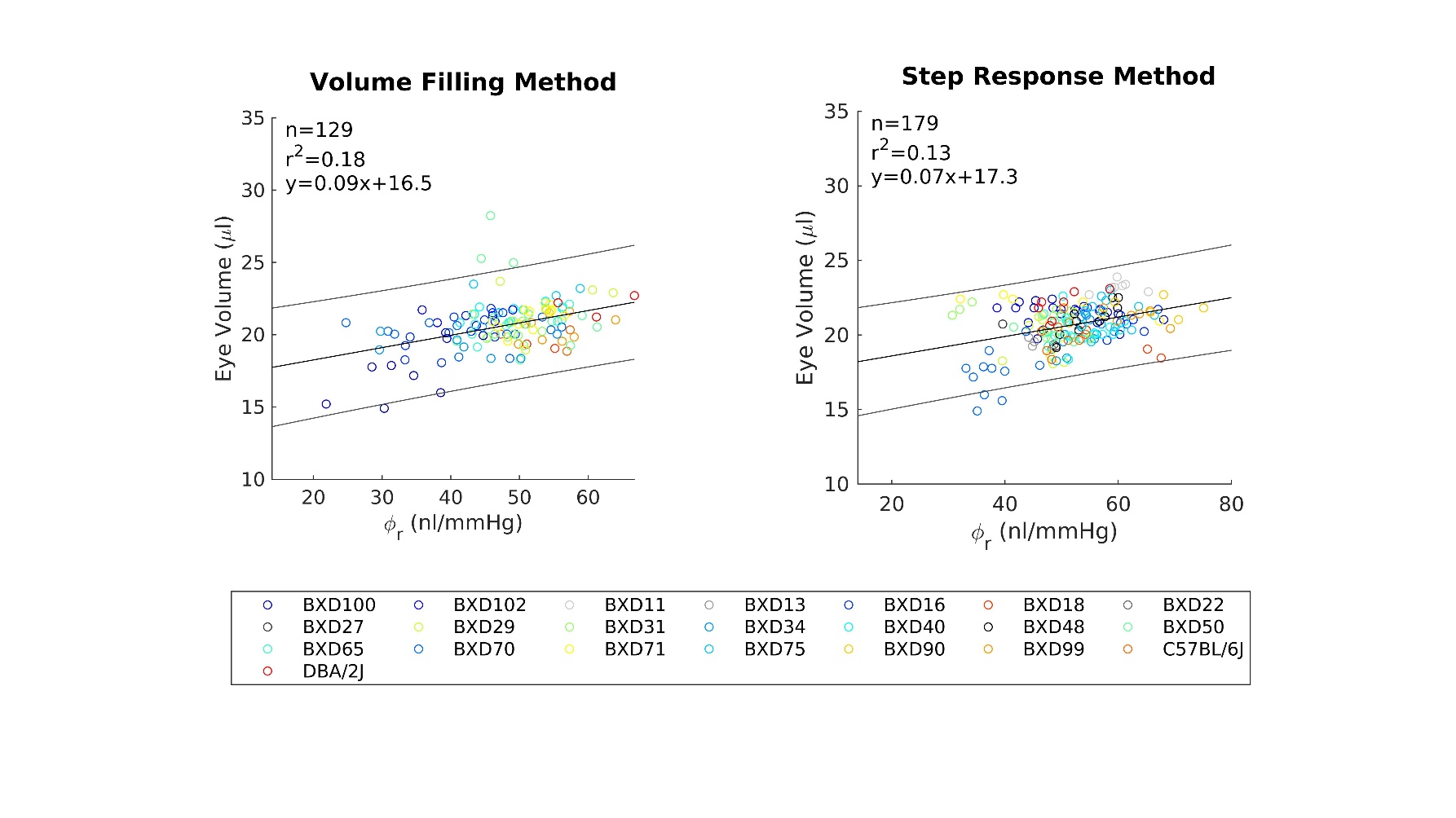


**B**

**A**

**Supplemental Figure 8:** Correlations between eye volume and ocular compliance as measured by the Volume Filling (A) and Step Response (B) methods. Data points represent the same eyes selected in the Volume Filling and Step Response data sets presented in Figure 1 and Supplemental Figures 1 & 2. Ocular compliance is plotted against the volume calculated from the measured weight of each individual eye. The color scheme is consistent with previous figures.
